# Antioxidant and Nutritional Properties of Domestic and Commercial Coconut Milk Preparations

**DOI:** 10.1155/2020/3489605

**Published:** 2020-08-01

**Authors:** Asiri N. Karunasiri, Mahendra Gunawardane, Chathuri M. Senanayake, Nimanthi Jayathilaka, Kapila N. Seneviratne

**Affiliations:** ^1^Department of Chemistry, Faculty of Science, University of Kelaniya, Kelaniya, Sri Lanka; ^2^Department of Microbiology, Faculty of Science, University of Kelaniya, Kelaniya, Sri Lanka

## Abstract

The aqueous extract of scraped coconut kernel is known as coconut milk. Coconut milk preparations are also commercially available in the form of desiccated powders or liquids. While these various coconut milk preparations are heavily used in cooking in the Asian countries as a major source of dietary fat, limited studies have been conducted on their chemical and nutritional composition. In this study, we have determined the chemical composition and nutritional effects of both domestic preparations of coconut milk and the commercially available counterparts. The results indicate that the phenolic compounds of all coconut milk preparations provide protection against oxidative damage on lipids and inhibit oxidative damage of both proteins and DNA. The lipid profiles are not significantly affected by the consumption of the three coconut milk preparations despite their different fat contents.

## 1. Introduction

Coconut milk is the aqueous extract of the solid endosperm (kernel) of coconut. In addition to the use for coconut oil extraction by wet process, coconut milk is directly used as a liquid medium in culinary applications to prepare dishes including meat and vegetable dishes. Domestic coconut milk is prepared by adding water to the scraped coconut kernel and mixing in a blender followed by filtering coconut milk through a strainer. Coconut milk is also available in the market in powder form and in liquid form. Some nutritional constituents and antioxidant properties of coconut milk have been reported. Coconut milk is an emulsion containing mainly lipid carbohydrates and proteins. It also contains several minor compounds including phenolic substances [1]. Antioxidant properties evaluated by ferric reducing power (FRAP) assay and 1,1-diphenyl-2-picrylhydrazyl (DPPH) assay indicate that coconut milk displays higher antioxidant properties than cow's milk [2]. Antioxidant properties of the methanolic extracts of the coconut kernel have also been tested with DPPH and 2,2-azino-bis(3-ethylbenzothiazoline-6-sulfonic acid) diammonium (ABTS) assays, as a function of maturity, and the antioxidant activities increased up to 190 days from the date of pollination and then decreased or remain unchanged [3]. Studies also indicate that the serum LDL levels decreased while the HDL levels increased in healthy subjects on a diet containing coconut milk [4].

Although few studies have reported the chemical and nutritional information of coconut milk, the chemical and nutritional properties of different coconut milk preparations have not been compared, and detailed studies on the protective effect of coconut milk antioxidants on oxidative stress-induced macromolecular damage have not been reported to the best of our knowledge. Harmful features of oxidative stress arise when oxidative forces exceed the antioxidant defense systems in biological systems. Oxidative stress is well known to be closely related to cancer, atherosclerosis, hypertension and diabetes mellitus. Phenolic antioxidants are well known to confer protection against oxidative damage in biological systems. In addition to interacting with reactive oxygen species (ROS) and neutralizing them, polyphenols inhibit the enzymes that are involved in the production of inflammation mediators, preventing inflammation and carcinogenesis induced by inflammation [5]. Lipids, proteins, and DNA are modified due to interactions with ROS, and the changes in these macromolecules can result in loss of physiological functioning which is also associated with the aging of post mitotic cells. Thus, such changes to macromolecules can be used as biomarkers of oxidative stress. Among lipid oxidation products, thiobarbituric acid reactive substances (TBARS) have been used as a marker of oxidative stress in obstructive sleep apnea [6]. Among other biomacromolecules, DNA is also damaged by oxidative stress. mt-DNA, which is more sensitive to oxidative stress damage, may alter mitochondrial gene expression in many human cancer cells [7]. Protein carbonyls that are formed due to oxidation of proteins are also stable biomarkers of oxidative stress.

Though coconut milk is used for cooking in many countries on a daily basis as a major source of dietary fat, the nutritional information of coconut milk has not sufficiently been investigated. The present study was conducted to compare basic chemical composition and the nutritional impact of domestic coconut milk, powdered coconut milk, and liquid coconut milk. The protective effect of the phenolic extracts of the three coconut milk preparations on the oxidative stress-induced macromolecular damage was evaluated using *Saccharomyces cerevisiae* (yeast) as a biological model. The nutritional effect of consumption of coconut milk based on the lipid profiles was evaluated using Wistar rats.

## 2. Materials and Methods

### 2.1. Sampling

Mature coconuts (12-14 months) collected from *Cocos nucifera* L., typica (tall type) coconut trees, characteristics of which have been reported, were used to prepare domestic coconut milk. This coconut cultivar was confirmed based on morphological characteristics as previously reported [8, 9]. For the analysis of commercial coconut milk samples, powdered coconut milk and liquid coconut milk with no added substances available in the market were used.

### 2.2. Preparation of Liquid Samples of Coconut Milk

To prepare domestic coconut milk, scraped coconut kernel (100 g) was added to distilled water (100 mL) and mixed using a kitchen blender (3 min). The resultant slurry was squeezed through cheesecloth to separate the milk portion, and the resultant liquid was used as domestic coconut milk (DCM). Commercially available coconut milk powder was dissolved in distilled water according to manufacturers' instructions and the resultant liquid was used as powdered coconut milk (PCM). Commercially available liquid coconut milk was directly used as liquid coconut milk (LCM) in the study based on the manufacturers' instructions. The densities of DCM, PCM, and LCM were not significantly different (1.0 ± 0.0 g/mL).

### 2.3. Preparation of Aqueous and Methanolic Extracts of Coconut Milk

DCM, PCM, and LCM samples were frozen, and the lipids were removed by centrifugation (1080 g, 10 min) to prepare aqueous extracts. To prepare the methanolic extract of DCM, aqueous extracts of DCM (1 mL) were mixed with methanol (1 mL) and chloroform (1 mL) and the mixture was vortexed (30 Hz, 1 min). Then, the sample was centrifuged (380 g, 10 min), and the top methanol/water layer was separated. The same procedure was used to prepare methanolic extracts of PCM and LCM.

### 2.4. Determination of Total Phenolic Content and Antioxidant Activity

Each aqueous extract of DCM, PCM, or LCM (15 *μ*L) was diluted by adding deionized water (135 *μ*L). Then, Folin-Ciocalteu reagent (6 *μ*L) and 25% Na_2_CO_3_ (15 *μ*L) were added to it. The mixture was diluted with deionized water (129 *μ*L) and incubated for 1 hour at room temperature. The absorbance of the sample was measured at 765 nm using a UV-visible spectrophotometer (Multiskan GO, Thermo Scientific, Finland) against a control sample with no added coconut milk aqueous extract. Gallic acid was used as the standard for the preparation of calibration curves. DPPH radical scavenging activity and ferric reducing power of the aqueous extracts of DCM, PCM, and LCM were evaluated as reported by Seneviratne and Kotuwegedara [10].

### 2.5. Total Sugar and Protein Content

Total sugar content of coconut milk was determined by the method of Ting [11]. The standard curve was prepared by using glucose with different concentrations. Protein content was determined by Coomassie dye-binding assay (Bradford assay). Standard series of protein samples with varying concentrations (0.15 mg/mL to 2 mg/mL) were prepared by using Bovine Serum Albumin (BSA). The negative controls were distilled water and methanol-water (50% *v*/*v*) for the aqueous extract of coconut milk and methanolic extract of coconut milk, respectively, with all other reagents.

### 2.6. Total Fat Content

The fat extraction method was adopted from a reported procedure used for the fat extraction from coconut water [12]. Coconut milk (DCM, PCM, or LCM) (10.00 mL) was mixed with hexane (10.00 mL) in a separatory funnel and shaken thoroughly, and the top hexane layer was collected. The extraction was continued with two fresh 10.0 mL portions of hexane. Then, hexane was removed, and the weight of the fat fraction was recorded.

### 2.7. Fatty Acid Composition

Methylation of fatty acids and the sample preparation for GC analysis was conducted according to a reported procedure [10]. A gas chromatograph (Shimadzu GC-2010 Plus, Japan) equipped with a capillary column which was RtxR-WAX (crossbond with PEG 30 m × 0.32 mm, i.d. 0.25 *μ*m) and a flame ionization detector (FID) were used for the analysis. The carrier gas was helium with the flow rate of 0.5 mL/min, and the analyses were performed on split mode (split ratio 100 : 1). Sample (1 *μ*L) was injected to the GC system. The temperatures of the injector and detector were 230°C and 250°C, respectively. A column temperature program of 130°C (3 min), 130°C to 210°C at 45°C/min, and 210°C (12 min) was used.

### 2.8. Identification of Phenolic Compounds

Phenolic compounds in the methanolic extracts of DCM, PCM, and LCM were identified and quantified by a previously reported method [13]. Briefly, prepared phenolic extract (20 *μ*L) was injected to the HPLC system. HPLC experiments were performed using an Agilent 1100-Infinity liquid chromatographic system (Agilent Technologies, Waldbronn, Germany) equipped with an Agilent 1200 diode array detector and a ZORBAX ECLIPSE Plus C18 column (Agilent Technologies, USA) (4.6 mm × 100 mm × 3.5 *μ*m particle size) maintained at room temperature. Methanol (A) and 1 × 10^−3^ mol dm^−3^ H_2_SO_4_ in deionized water (B) were used as the mobile phase. The flow rate was 0.5 mL min^−1^, and the total running time was 90 min. The elution gradient began with 5% A and 95% B. From 0 to 15 min, A was increased to 10%, and this composition (10% A and 90% B) was continued up to 30 min. Then, A was increased to 20% from 30 to 40 min, to 30% from 40 to 50 min, to 40% from 50 to 60 min, to 50% from 60 to 70 min, and to 60% from 70 to 80 min, and this composition (60% A and 30% B) was continued for 10 min. Phenolic compounds were detected at 280 nm and identified by comparison of the retention times and UV spectra of authentic standards.

### 2.9. Inhibition of Macromolecular Damage by Coconut Milk Antioxidants

#### 2.9.1. Yeast Strain and Cultivation

*Saccharomyces cerevisiae* (DMS 1333; ATCC 9763) was obtained from DSMZ—German Collection of Microorganisms and Cell Cultures, and the culture was stored and maintained in a glycerol stock (15%) at -80°C. The yeast cultures for the experiments were grown in YPDA (yeast extract 10 g L^−1^, peptone 20 g L^−1^, dextrose 20 g L^−1^, and agar 15 g L^−1^) and incubated at room temperature. Broth cultures in YPD (yeast extract 10 g L^−1^, peptone 20 g L^−1^, and dextrose 20 g L^−1^) were incubated at room temperature at 250 rpm. Overnight cultures at room temperature at 250 rpm inoculated from the glycerol stocks were used to set up fresh cultures in the log phase for the antioxidant activity assays according to a previously published method with some modifications [14]. The fresh cultures were obtained by 1 : 40 dilution of the overnight culture into sterile YPD medium and incubation at room temperature at 250 rpm for 3-4 hours until the cultures reach an OD of 0.5 at 620 nm.

#### 2.9.2. Treatment of Yeast Cell Suspensions with Antioxidants

Yeast cell suspensions were treated with phenolic extracts of coconut milk solutions based on a reported method [15]. Methanolic extracts were used to make the stock antioxidant solutions. For example, methanolic extracts of DCM were evaporated, the residue was dissolved in distilled water to obtain the stock phenolic extract (20.0 mg mL^−1^), and the phenolic extract was sterilized by filtering through a 0.45 *μ*m sterile filter. The procedure was repeated for methanolic extracts of PCM and LCM. For every experiment, yeast cultures at 0.5 OD maintained in YPD broth were treated overnight with the abovementioned stock phenolic extract at a 1 : 40 dilution in 1 mL of YPD broth culture to obtain 0.5 mg mL^−1^ final concentration. The control sample was treated with the same volume of distilled water instead of the phenolic extract. Sterile conditions were maintained throughout all the steps of the experiments.

#### 2.9.3. Oxidative Stress Induction of Yeast

YPD broth cultures of yeast were used to induce the oxidative stress by H_2_O_2_ after treating with phenolic extract at 0.5 mg mL^−1^ [14]. The broth cultures were centrifuged (100 g, 3 min) to harvest yeast cell pellets, and the supernatants were removed along with the antioxidants. Then, 1x PBS (1 mL) was added to resuspend the pellets to wash residual antioxidants. Samples were centrifuged (100 g, 3 min) again and supernatants were removed. Then, the cell pellets were resuspended in 1x PBS (1 mL), and 15 mM FeCl_2_ was added to reach 150 *μ*M FeCl_2_. The mixture was incubated for 30 min at room temperature at 250 rpm followed by induction of oxidative stress with 2 mM H_2_O_2_ (this concentration was deemed to be the maximum H_2_O_2_ concentration that does not significantly affect the viability of the cells). The samples were incubated for 1 hour at room temperature at 250 rpm. The control sample contained the same composition except water in the place of H_2_O_2_.

#### 2.9.4. Yeast Survival Assay

The oxidative stress-induced macromolecular damage was assessed under the conditions that do not significantly affect the yeast cell viability according to a previously reported method [14]. The yeast cells were diluted 1 : 4000 with 1x PBS, and 25 *μ*L was plated on YPDA plates followed by incubation for 48 hours at room temperature to obtain viable cell colony counts. The percentage survival of yeast was calculated based on the number of colony-forming units (CFUs) according to the following formula.

(1) $$\begin{array}{c} \text{The percentage survival of yeast}=\frac{\text{CFU of sample}\times 100\%}{\text{CFU control}}. \end{array}$$

#### 2.9.5. Inhibition of Protein Carbonylation

Inhibition of protein carbonyl formation by coconut milk antioxidants was assessed according to a reported method [16]. Oxidative stress-induced yeast cells (1 mL) were harvested by centrifugation (380 g, 10 min). The resultant pellet was resuspended in 1x PBS (1 mL) to remove H_2_O_2_ and stop oxidative damage and centrifuged (380 g, 10 min) to harvest the cell pellet. The cells were lysed in 100 *μ*L 1x lysis buffer (1% Tween 20, 0.1% SDS, and 50 mM Tris HCl, pH 7) with vortexing (40 Hz, 10 seconds). TCA (30% *w*/*v*, 250 *μ*L) was added to the mixture to precipitate the protein and vortexed (40 Hz, 10 seconds). Then, the mixture was centrifuged (300 g, 3 min) and protein pellets were separated. 2,4-DNPH (10 mM in 2 M HCL, 250 *μ*L) was added to the pellets, mixed, and incubated at room temperature for 60 min in the dark. Then, TCA (20%, 250 *μ*L) was added and centrifuged (11000 g, 3 min). The pellet was separated, washed three times with ethanol : ethyl acetate mixture (1 : 1 *v*/*v*) and incubated for 10 min. Then, the mixture was centrifuged (11000 g, 3 min) and the pellet was separated. Guanidine solution (6 M, 300 *μ*L) was added to the pellet to reconstitute the precipitated proteins and centrifuged (11000 g, 3 min). The absorbance of the supernatant was measured at 370 nm by using a UV-visible spectrophotometer. The protein carbonyl content was calculated using the following formula.

(2) $$\begin{array}{c} \text{Protein carbonyl}\left(\text{nmol}/\text{mL}\right)=\frac{A_{s}-A_{c}}{X}\times \frac{300}{200}, \end{array}$$

*X* is the extinction coefficient for DNPH (0.011 *μ*M^−1^), *A*_*s*_ is the absorbance of the sample, and *A*_*c*_ is the absorbance of the blank (guanidine solution).

#### 2.9.6. Inhibition of Mitochondrial DNA (mt-DNA) Damage

mt-DNA damage was assessed according to a previously reported method with modifications [17]. Yeast colonies grown on YPDA plates after induction of oxidative stress were copied onto the YPGA (yeast extract 1%, Bacto-Peptone 2%, glycerol 2%, ethanol 2%, and agar 2%) plates and incubated for 48 hours at room temperature. The percentage of survival based on the CFU in YPGA was used to assess the percentage of cells with mt-DNA damage according to following equation.

(3) $$\begin{array}{c} \text{mt}‐\text{DNA damaged cells}\left(\%\right)=\left(\frac{\text{CFU in YPDA}-\text{CFU in YPGA}}{\text{CFU in YPDA}}\right)*100. \end{array}$$

#### 2.9.7. Inhibition of Lipid Peroxidation

Thiobarbituric acid reactive substances (TBARS) were measured using linoleic acid as an external source of polyunsaturated fatty acids according to a previously published method with some modifications [14, 18]. Linoleic acid was homogenized in Tween 20 and H_2_O mixture (1 : 2000 *v*/*v*, respectively) at room temperature for 5 min at 250 rpm. The homogenate was added to the overnight cultures of antioxidant-treated yeast cells at an end concentration of 0.03 mg/mL and incubated for 4 hrs at room temperature at 250 rpm prior to the induction of oxidative stress. The control sample contained the linoleic acid homogenate, FeCl_2_, and H_2_O instead of H_2_O_2_. The cells were harvested by centrifugation (380 g, 10 min) and washed with 1x PBS (1 mL). Resultant pellets were lysed as stated above, and 1 mL thiobarbituric acid (TBA) (15% TCA, 0.8% TBA in 0.25 N HCl) was added and mixed well. BHT (0.2%, 8 *μ*L) was added, and samples were heated for 15 min at 95°C. The mixture was allowed to cool and centrifuged at 380 g for 20 min. The absorbance of the supernatant was measured at 532 nm by Multiskan GO spectrophotometer (Thermo Scientific). The standard curve was prepared by using malondialdehyde.

### 2.10. Animal Studies

#### 2.10.1. Feeding Rats

Ethical approval for the animal study using a rat model was obtained from the Ethics Review Committee of the Faculty of Medical Sciences, University of Sri Jayewardenepura (Application number: 25/16). Feeding experiments were conducted as reported [19]. Briefly, seven-week-old male Wistar rats (weighing 240-300 g) were selected from the Medical Research Institute, Colombo, Sri Lanka. The animals were housed in cages in a room maintained at 25 ± 1°C with a 12 h light and dark cycle. Prior to the commencement of the experiment, rats were acclimatized to the basal diet for 6 days. Then, the rats were randomly assigned into experimental groups (7 rats/group).

The control group was fed with the basal diet. The second, third, and fourth groups of rats were fed with the test diets containing 12 mL each of DCM, PCM, and LCM, respectively, per kg of basal diet. The study was continued for 150 days. Free access to water and diet was provided throughout the experimental period. Rats were monitored daily. Body weight and feed intake were measured weekly.

#### 2.10.2. Analysis of Serum Lipid Profiles

The rats were fasted for 10-12 h prior to drawing blood on day 0, 30 days, 90 days, 120 days, and 150 days after feeding the experimental diets. Blood (500 *μ*L) was drawn from the tail vein and collected into plain tubes and centrifuged to harvest serum. Serum total cholesterol (TC), serum high-density lipoprotein (HDL) cholesterol, and serum triglyceride (TG) levels were determined using the test kit provided by G Cell (Beijing Strong Biotechnologies, Inc., China). The method given in the test kit was followed without any modifications. Serum low-density lipoprotein (LDL) cholesterol was determined using the Friedewald equation (LDL = TC–HDL − (TG/5)).

#### 2.10.3. Dissection and Harvesting Tissues from Animals

At the end of the feeding experiments, the animals were subjected to barbiturate euthanasia. Body weight was measured before dissecting the animals. The liver and heart were harvested, and the liver size, liver weight, and heart weight were recorded. Pericardium thickness was measured using a Vernier caliper. Average weight gain during the study period was calculated according to the following formula.

(4) $$\begin{array}{c} \text{Average body weight gain}=\text{average final body weight}-\text{average initial body weight}. \end{array}$$

### 2.11. Statistical Analysis

All experiments were run in triplicate, and biological replicates were carried out unless otherwise indicated. A two-sample *t*-test or one-way ANOVA was carried out for the determination of significant differences (*p* ≤ 0.05) between the means. Data were analyzed using Minitab (Version 17 for Windows).

## 3. Results and Discussion

### 3.1. Basic Nutritional Composition and Antioxidant Activity

Basic nutrient composition in the aqueous DCM, PCM, and LCM given in Table 1 indicates that there is no significant difference in total phenol content, total sugar contents, and power reduction of DCM, PCM, and LCM while the protein content in LCM was significantly lower compared to that in DCM and PCM (*p* < 0.05; *n* = 27). In contrast to the power reduction, DPPH radical scavenging activity showed significantly lower values in PCM and LCM compared to DCM, despite the fact that no significant differences were observed among the corresponding phenolic antioxidant contents of the aqueous extracts. Such discrepancies could be due to some differences of the other nutrient contents as reported [20]. For example, proteins have been reported to display radical scavenging activity as determined by the DPPH assay due to the antioxidant activity of sulfur-containing amino acids such as methionines and cysteines [21]. Therefore, methanolic extracts of DCM, PCM, and LCM were also prepared by removing proteins using organic extraction with chloroform to assess the DPPH radical scavenging activity. Complete removal of proteins by organic extraction was confirmed by the Bradford assay. There were no significant differences among the percentage DPPH radical scavenging activities of the methanolic extracts from the three different coconut milk preparations after protein removal (DCM: 34.6 ± 0.2, PCM: 32.2 ± 10.7, and LCM: 31.2 ± 12.9). Therefore, proteins may have contributed to the significant differences in antioxidant activity of the aqueous extracts of coconut milk preparations as assessed by DPPH assay. In addition, total fat contents of the three coconut milk samples were significantly different (*p* < 0.05). Basic nutritional parameters for DCM, PCM, and LCM are compared with reported values for cow's milk [22–24], goat's milk [23, 25, 26], and soy milk [24, 27, 28] in Table 1. However, basic nutritional composition drastically changes with the diet, environmental conditions, season, and breed in cow's milk and goat's milk.

The composition of major fatty acids present in DCM, PCM, and LCM is given in Table 2. The variation of fatty acid composition in DCM, PCM, and LCM is well within the reported range of the fatty acid composition of coconut fat [29]. Table 2 also compares the composition of major fatty acids in DCM, PCM, and LCM with the compositions of those fatty acids in cow's milk [30], goat's milk [31], and soy milk [32]. Except for palmitic acid, major fatty acids in cow's milk, goat's milk, and soy milk are longer chain fatty acids.

### 3.2. Identification of Phenolic Compounds

**Figure 1:**
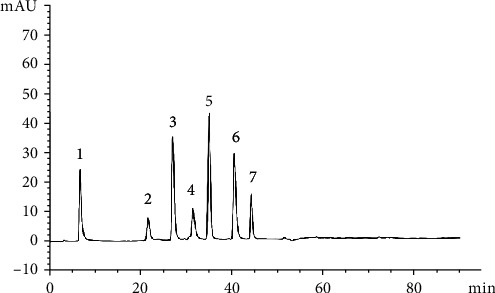
HPLC chromatogram of the phenolic extracts of DCM (1) gallic acid, (2) chlorogenic acid, (3) parahydroxybenzoic acid, (4) caffeic acid, (5) vanillic acid, (6) syringic acid, and (7) ferulic acid.

The phenolic compounds in coconut milk originate from the brown coconut testa and white coconut kernel. These phenolic substances are thermally stable at cooking temperatures [33]. Therefore, antioxidant properties of the phenolic substances present in coconut milk may be retained during cooking.  Figure 1 shows the HPLC chromatogram of the phenolic extract of DCM. Phenolic compounds present in PCM and LCM are also similar according to the HPLC chromatograms of PCM and LCM. HPLC chromatograms indicate that there are seven phenolic compounds present in coconut milk. Comparison of the quantities of phenolic substances present in DCM, PCM, and LCM indicates that the phenolic compositions for most phenolic compounds are similar in the three coconut milk types.

### 3.3. Antioxidant Activity under Stressed Conditions

**Figure 2:**
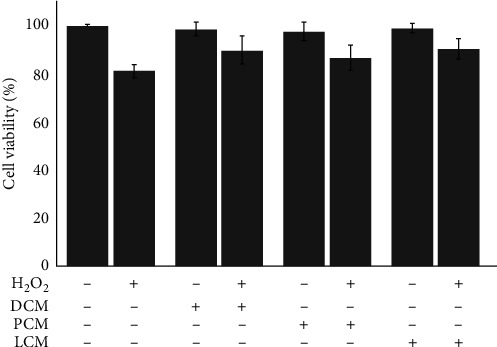
Protective effect of phenolic extracts of DCM, PCM, and LCM on the oxidative stress-induced damage of yeast cells given as percentage viability (*n* = 4).

Antioxidant potential of plant extracts is usually evaluated by chemical methods such as DPPH radical scavenging activity and reducing power assays. However, antioxidant activities evaluated in chemical models may not always correlate in biological systems [34]. Therefore, it is also important to use biological models to investigate antioxidant activities of phenolic extracts to get a clear idea about the true antioxidant potential and to further confirm the results obtained in chemical systems. Yeast cells were used in the present study as a biological model to test the antioxidant potential of the phenolic compounds of DCM, PCM, and LCM. Yeast cell suspensions were pretreated with phenolic antioxidants of DCM, PCM, and LCM followed by the induction of oxidative stress using H_2_O_2_ after removing the antioxidants. The oxidative stress was induced at a concentration at which the cell viability is above 80% to assess the effect of the antioxidants on oxidative stress-induced macromolecular damage (Figure 2). The antioxidant-treated cells have a significantly higher (*p* < 0.05; *n* = 4) percentage of viable cells under induced stress conditions compared to untreated control cells suggesting that coconut milk antioxidants provide protection against H_2_O_2_-induced cell death. However, there is no significant difference among survival rates of yeast in the samples treated with different coconut milk antioxidant extracts followed by H_2_O_2_-induced oxidative stress.

To evaluate the effect of coconut milk antioxidants on the inhibition of lipid peroxidation in yeast cells, levels of TBARS were analyzed after subjecting yeast cells to oxidative stress. Polyunsaturated fatty acids undergo oxidation to produce TBARS. Yeast does not normally produce polyunsaturated fatty acids though it can utilize exogenously provided polyunsaturated fatty acids [14]. Antioxidants play an important role *in vivo* in protecting cells from oxidative damage to polyunsaturated fatty acids. Therefore, effect of antioxidants on the inhibition of TBARS formation in yeast cells can be evaluated by providing polyunsaturated fatty acids to the medium.

Similarly, effect of antioxidants on the inhibition of protein carbonyl formation due to oxidative damage in yeast cells was evaluated.  Table 3 shows that both TBARS and protein carbonyl levels in yeast lysates from cultures pretreated with the phenolic extracts of DCM, PCM, and LCM are significantly lower (*p* < 0.05; *n* = 4) compared to the TBARS and protein carbonyl levels of H_2_O_2_-stressed yeast lysates without pretreatment with phenolic antioxidants while phenolic extracts of both DCM- and PCM-treated samples maintained lower protein carbonyl and TBARS levels compared to yeast lysates treated with the phenolic extracts of LCM.

Reactive oxygen species such as hydrogen peroxide causes lesions in DNA [35]. mt-DNA shows a high susceptibility towards oxidative damage. Therefore, DNA damage in yeast cells can be assessed based on the fact that growth of yeast cells on YPG (glycerol-containing) medium requires mitochondrial respiration, while growth on YPD (glucose-containing) medium is possible without it. As such, the DNA damage assay in the present study is based on the assumption that yeast cells with damaged mt-DNA are unable to utilize glycerol as the source of carbon and energy [17]. The mt-DNA damage assay indicates that phenolic antioxidants have a statistically significant (*p* < 0.05; *n* = 4) protective effect against oxidative stress-induced DNA damage compared to stressed yeast cells that were not pretreated with antioxidants as indicated by the percentage of respiratory-deficient cells (Table 3).

### 3.4. Effect of Different Coconut Milk Preparations on Serum Lipid Profiles

Some studies indicate that saturated fat increases the serum levels of cholesterol. However, a recent report indicates that medium chain fatty acids decrease serum cholesterol levels via reduction of bile absorption [36]. As indicated in Table 2, major fatty acids of coconut milk are medium chain fatty acids.  Table 4 shows the effect of feeding Wistar rats with DCM-, PCM-, and LCM-containing diets on serum total cholesterol, LDL, and HDL levels. There is no statistically significant increase of total cholesterol, LDL, and HDL levels over 150 days of feeding DCM, PCM, and LCM compared to the control group (*n* = 7). Serum triglyceride levels increased significantly in rats fed with control or coconut milk fed rats over long-term feeding of the diets (*p* < 0.05). However, there is no significant difference in TG levels in rats fed with diets containing DCM, PCM, and LCM compared to control rats during the study period. There is a higher saturated fat content in the DCM-, PCM-, and LCM-containing diets compared to the control diet. However, increased fat content of the test diets compared to the control diet has not altered lipid profiles significantly. This observation may be due to the presence of medium chain saturated fats in coconut milk.

At the conclusion of the dietary intervention, weight gain, heart and liver size, heart and liver weight, and pericardium thickness were measured to assess the impact of the test diets on the physical parameters that have been associated with the risk of developing cardiovascular disease due to deposition of fat. There is no significant difference observed in final body weight, weight gain, liver size, liver weight, heart weight, and pericardium thickness in rats in the studied groups.

## 4. Conclusions

Phenolic substances of coconut milk may protect macromolecules such as lipids, proteins, and DNA against oxidative damage in living systems. A considerable difference in the basic nutrient composition of DCM, PCM, and LCM could be observed only for fat content. However, serum lipid profiles, body weight, average weight gain, liver size, liver weight, heart weight, and pericardium thickness are not affected by the differences in fat contents of DCM, PCM, and LCM compared to a control diet without coconut milk, suggesting that consumption of coconut milk may not affect these health parameters. However, more studies with larger quantities of coconut milk in the diet are necessary to decide the healthy limit of coconut milk in the diet.

## Figures and Tables

**Table 1:** Basic nutrient composition of DCM, PCM, and LCM and reported values for cow's milk, goat's milk, and soy milk.

	DCM	PCM	LCM	Cow's milk	Goat's milk	Soya milk
Total phenol content (mg/L)	8.21 ± 0.13^a^	8.23 ± 0.14^a^	8.23 ± 0.12^a^	13.31 [22]	87.9 [25]	61.4 [27]
Protein (%)	5.83 ± 0.48^a^	4.53 ± 0.23^b^	4.13 ± 0.19^b^	2.82 [23]	3.48 [23]	4.50 [28]
Fat (%)	3.11 ± 0.16^b^	1.83 ± 0.70^c^	3.99 ± 0.40^a^	3.42 [23]	5.23 [23]	4.30 [28]
Carbohydrates (%)	2.21 ± 0.01^a^ (total sugars)	1.94 ± 0.26^a^ (total sugars)	2.10 ± 0.59^a^ (total sugars)	4.47 [23] (lactose)	4.11 [23] (lactose)	10.00 [28] (total carbohydrates)
DPPH (%)	54.92 ± 3.32^a^	44.08 ± 1.17^b^	43.32 ± 0.06^b^	8.70 [24]	56.55 [26]	33.51 [24]
Reducing power (%)	234.5 ± 2.8^a^	231.7 ± 12.1^a^	228.4 ± 9.1^a^	∗	∗	∗

Letters a, b and c were used to compare statistical significance (*p* ≤ 0.05) in the same row (*n* = 27). ^∗^Data not available under comparable conditions and units.

**Table 2:** Fatty acid composition of DCM, PCM, and LCM and reported values for cow's milk, goat's milk, and soy milk.

Fatty acid composition (%)	DCM	PCM	LCM	Cow's milk [30]	Goat's milk [31]	Soya milk [32]
Caprylic acid (C8)	7.45 ± 0.12^b^	6.64 ± 0.51^c^	8.34 ± 0.05^a^	—	2.7	—
Capric acid (C10)	6.84 ± 0.05^a^	6.70 ± 0.04^a^	6.93 ± 0.08^a^	0.03	10.0	—
Lauric acid (C12)	55.52 ± 0.02^a^	51.47 ± 0.80^b^	55.01 ± 0.02^a^	1.65	5.7	—
Myristic acid (C14)	22.08 ± 0.07^b^	24.34 ± 0.73^a^	21.66 ± 0.02^b^	0.52	11.7	0.07
Palmitic acid (C16)	8.01 ± 0.07^b^	10.84 ± 0.54^a^	8.05 ± 0.14^b^	34.02	26.3	10.7

Letters a, b, and c were used to compare statistical significance (*p* ≤ 0.05) in the same row (*n* = 4).

**Table 3:** Levels of protein carbonyls, TBARS in yeast cell lysates, and respiratory-deficient cells treated with different coconut milk antioxidants.

	Unstressed	Stressed			
		Control^∗^	DCM	PCM	LCM
Protein carbonyl (nmol/mL)	9.1 ± 0.3^c^	15.2 ± 0.3^a^	14.2 ± 0.1^b^	14.0 ± 0.1^b^	14.8 ± 0.1^a^
TBARS (*μ*g/mL)	0.00 ± 0.00^d^	0.05 ± 0.00^a^	0.02 ± 0.00^b^	0.02 ± 0.0^b^	0.02 ± 0.00^b^
Respiratory-deficient cells (%)	0.0 ± 0.0^c^	10.0 ± 2.8^a^	5.0 ± 4.2^b^	3.0 ± 1.4^b^	7.0 ± 1.4^a^

Letters a, b, and c were used to compare statistical significance (*p* ≤ 0.05) in the same row (*n* = 4). ^∗^Stress induced without prior exposure to antioxidants.

**Table 4:** Serum lipid profiles of rats fed with diets containing DCM, PCM, and LCM.

Lipid parameter	Day	Value (mg/dL)			
		Control	DCM	PCM	LCM
Total cholesterol	0	68.83 ± 3.14^aq^	71.16 ± 4.03^aq^	70.48 ± 5.59^aq^	72.30 ± 5.63^aq^
	30	71.17 ± 6.59^bq^	78.42 ± 5.41^ap^	71.73 ± 3.83^bq^	79.03 ± 6.43^aq^
	90	75.36 ± 3.04^ap^	81.03 ± 9.60^ap^	76.86 ± 9.30^ap^	83.12 ± 9.37^ap^
	120	75.12 ± 2.44^ap^	79.25 ± 5.34^ap^	77.75 ± 4.53^ap^	80.32 ± 5.65^ap^
	150	77.42 ± 6.97^bp^	79.25 ± 4.36^bp^	80.32 ± 5.21^bp^	81.00 ± 3.46^ap^

HDL cholesterol	0	27.81 ± 4.64^ap^	30.14 ± 7.48^ap^	28.51 ± 3.72^ap^	30.60 ± 3.51^ap^
	30	25.05 ± 4.27^ap^	27.86 ± 5.66^ap^	25.79 ± 5.21^ap^	28.97 ± 1.63^ap^
	90	27.08 ± 7.51^ap^	27.08 ± 3.39^ap^	29.66 ± 6.98^ap^	30.84 ± 7.08^ap^
	120	27.08 ± 4.01^ap^	27.75 ± 8.10^ap^	28.43 ± 3.16^ap^	29.98 ± 3.38^ap^
	150	25.42 ± 5.46^ap^	31.59 ± 5.09^ap^	26.73 ± 5.87^ap^	30.81 ± 3.79^ap^

LDL cholesterol	0	17.00 ± 5.17^ap^	16.98 ± 6.76^ap^	16.14 ± 3.69^ap^	15.58 ± 4.76^ap^
	30	22.24 ± 8.36^ap^	20.74 ± 6.79^ap^	16.25 ± 6.24^ap^	16.57 ± 7.94^ap^
	90	21.59 ± 8.19^ap^	24.89 ± 7.23^ap^	20.56 ± 7.41^ap^	23.29 ± 9.98^ap^
	120	17.34 ± 4.60^ap^	20.65 ± 7.50^ap^	20.15 ± 5.09^ap^	20.54 ± 6.34^ap^
	150	20.24 ± 6.41^ap^	17.61 ± 5.57^ap^	21.27 ± 8.31^ap^	20.19 ± 3.07^ap^

Triglycerides	0	120.06 ± 8.05^aq^	120.20 ± 3.64^aq^	125.90 ± 12.49^aq^	128.79 ± 15.60^aq^
	30	119.39 ± 12.72^aq^	149.12 ± 13.93^bp^	148.49 ± 15.63^bp^	167.46 ± 20.11^bp^
	90	137.85 ± 14.93^aq^	142.76 ± 14.39^ap^	130.73 ± 8.27^aq^	145.10 ± 10.73^ap^
	120	162.38 ± 14.88^ap^	150.38 ± 17.92^ap^	145.86 ± 14.95^ap^	149.98 ± 18.05^ap^
	150	149.46 ± 11.61^ap^	160.71 ± 14.82^ap^	159.28 ± 17.79^ap^	150.78 ± 12.37^ap^

Letters a, b, and c were used to compare statistical significance (*p* ≤ 0.05) in the same row. Letters p and q were used to compare statistical significance (*p* ≤ 0.05) in the same column within one lipid parameter. Each data point represents the mean ± standard deviation of seven replicates.

## Data Availability

The data used to support the findings of this study are included within the article and in the supplementary information files provided with the paper.

## Supplementary Materials

Supplementary Materials Supplementary Figure 1, Supplementary Table 1, and Supplementary Table 2. Supplementary Figure 1: gives the HPLC chromatograms of the phenolic substances of PCM and LCM. Supplementary Table 1: gives the quantities of phenolic compounds of DCM, PCM, and LCM. Supplementary Table 2: gives the body, liver, and heart characteristics of rats fed with different types of coconut milk diets.
