# Supplementary material for: Antioxidant and Nutritional Properties of Domestic and Commercial Coconut Milk Preparations
Source: Int J Food Sci. 2020 Aug 1;2020:3489605. doi: 10.1155/2020/3489605 (PMC7422486; doi:10.1155/2020/3489605)
Supplement: Supplementary Materials — Supplementary Figure 1, Supplementary Table 1, and Supplementary Table 2. Supplementary Figure 1: gives the HPLC chromatograms of the phenolic substances of PCM and LCM. Supplementary Table 1: gives the quantities of phenolic compounds of DCM, PCM, and LCM. Supplementary Table 2: gives the body, liver, and heart characteristics of rats fed with different types of coconut milk diets. [file 3489605.f1.zip › 3489605.f1/Supplementary Figure 1.pdf]

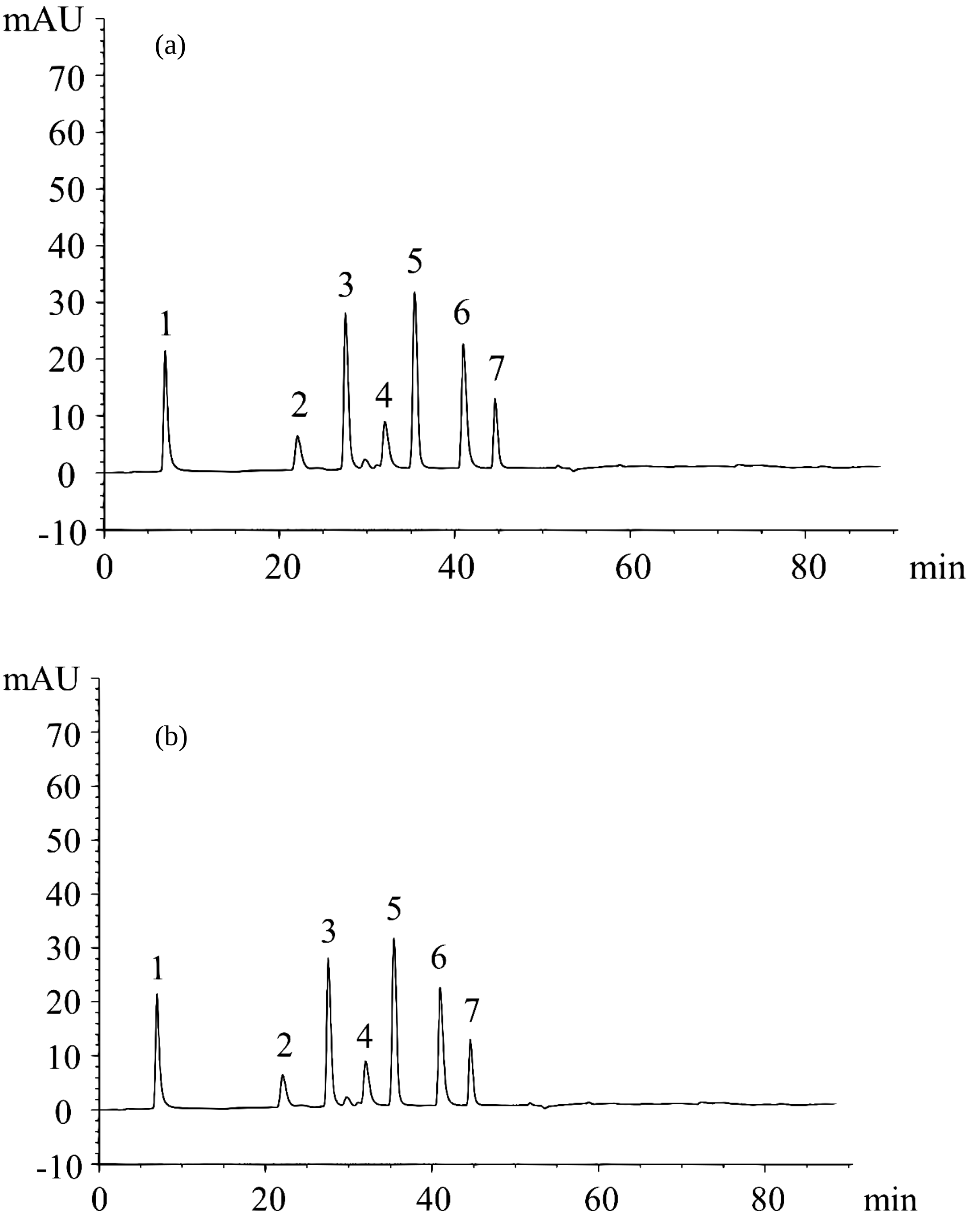

Supplementary Figure 1 (a) HPLC chromatogram of the phenolic substances of PCM (b) HPLC chromatogram of the phenolic extracts of LCM (1) gallic acid, (2) chlorogenic acid, (3) para-hydroxybenzoic acid, (4) caffeic acid, (5) vanillic acid, (6) syringic acid, (7) ferulic acid
