# Supplementary material for: Antioxidant and Nutritional Properties of Domestic and Commercial Coconut Milk Preparations
Source: Int J Food Sci. 2020 Aug 1;2020:3489605. doi: 10.1155/2020/3489605 (PMC7422486; doi:10.1155/2020/3489605)
Supplement: Supplementary Materials — Supplementary Figure 1, Supplementary Table 1, and Supplementary Table 2. Supplementary Figure 1: gives the HPLC chromatograms of the phenolic substances of PCM and LCM. Supplementary Table 1: gives the quantities of phenolic compounds of DCM, PCM, and LCM. Supplementary Table 2: gives the body, liver, and heart characteristics of rats fed with different types of coconut milk diets. [file 3489605.f1.zip › 3489605.f1/Supplementary Table 1.pdf]

Supplementary Table 1. Quantities of phenolic compounds present in DCM, PCM and LCM.

Compound name	Concentration (μg/100mL)		
	DCM	PCM	LCM
Gallic acid	1.51±0.32[a]	0.90±0.18[b]	1.01±0.07[b]
Chlorogenic acid	1.36±0.38[a]	0.90±0.09[b]	0.94±0.05[b]
Para-hydroxybenzoic acid	0.10±0.03[a]	0.07±0.02[a]	0.08±0.01[a]
Caffeic acid	0.81±0.22[a]	0.54±0.13[a]	0.59±0.08[a]
Vanillic acid	0.72±0.19[a]	0.53±0.16[a]	0.61±0.11[a]
Syringic acid	1.36±0.34[a]	1.02±0.30[a]	1.11±0.19[a]
Ferulic acid	0.50±0.13[a]	0.35±0.09[a]	0.38±0.06[a]

Letters a and b were used to compare statistical significance ($p \leq 0.05$) in the same row.
