# Supplementary material for: Antioxidant and Nutritional Properties of Domestic and Commercial Coconut Milk Preparations
Source: Int J Food Sci. 2020 Aug 1;2020:3489605. doi: 10.1155/2020/3489605 (PMC7422486; doi:10.1155/2020/3489605)
Supplement: Supplementary Materials — Supplementary Figure 1, Supplementary Table 1, and Supplementary Table 2. Supplementary Figure 1: gives the HPLC chromatograms of the phenolic substances of PCM and LCM. Supplementary Table 1: gives the quantities of phenolic compounds of DCM, PCM, and LCM. Supplementary Table 2: gives the body, liver, and heart characteristics of rats fed with different types of coconut milk diets. [file 3489605.f1.zip › 3489605.f1/Supplementary Table 2.pdf]

Supplementary Table 2: Body, liver and heart characteristics of rats fed with different types of coconut milk diets

Group	Average final Body weight (g)	Average weight gain (g)	Liver size (cm)	Liver weight (g)	Heart weight (g)	Pericardium thickness (mm)
Control	384.29±29.92*	112.86±11.13*	2.1±0.1*	14.31±0.31*	1.14±0.08*	1.1±0.1*
DCM	380.00±33.17*	115.71±29.36*	2.0±0.1*	14.25±1.51*	1.15±0.11*	1.0±0.3*
PCM	361.43±38.48*	112.86±31.47*	2.0±0.2*	14.34±1.24*	1.24±0.13*	1.1±0.3*
LCM	360.00±25.17*	108.57±18.64*	2.0±0.1*	14.33±1.05*	1.16±0.12*	1.0±0.2*

*Denotes no significant difference in the same column. Each data point represents the mean±SD (n=7).
